# Differential Gene Expression in Peripheral White Blood Cells with Permissive Underfeeding and Standard Feeding in Critically Ill Patients: A Descriptive Sub-study of the PermiT Randomized Controlled Trial

**DOI:** 10.1038/s41598-018-36007-w

**Published:** 2018-12-20

**Authors:** Yaseen M. Arabi, Mohammed Al-Balwi, Ali H. Hajeer, Dunia Jawdat, Musharaf Sadat, Hasan M. Al-Dorzi, Hani Tamim, Lara Afesh, Walid Almashaqbeh, Haitham Alkadi, Deemah Alwadaani, G. K. UdayaRaja, Ibrahim B. Al Abdulkareem, Abdulaziz Al-Dawood

**Affiliations:** 1 College of Medicine, King Saud bin Abdulaziz University for Health Sciences, King Abdullah International Medical Research Center, Intensive Care Department, King Abdulaziz Medical City, Riyadh, Saudi Arabia; 2 Department of Clinical Laboratory, King Saud bin Abdulaziz University for Health Sciences, King Abdullah International Medical Research Center, King Abdulaziz Medical City, Riyadh, Saudi Arabia; 3 Department of Pathology and Laboratory Medicine, King Saud bin Abdulaziz University for Health Sciences, King Abdullah International Medical Research Center, King Abdulaziz Medical City, Riyadh, Saudi Arabia; 4 Cord Blood Bank, King Abdullah International Medical Research Center, King Saud bin Abdulaziz University for Health Sciences, King Abdulaziz Medical City, Riyadh, Saudi Arabia; 5 Department of Internal Medicine, American University of Beirut Medical Center, Beirut, Lebanon; 6 Medical Genomics, King Abdullah International Medical Research Center, King Saud bin Abdulaziz University for Health Sciences, King Abdulaziz Medical City, Riyadh, Saudi Arabia; 7 Bioinformatics department, Integrated Gulf Biosystems, Riyadh, Saudi Arabia; 8 Pathology and Laboratory Medicine, King Abdullah University Hospital, Princess Nourah Bint Abdulrahman university, Riyadh, Saudi Arabia

## Abstract

The effect of short-term caloric restriction on gene expression in critically ill patients has not been studied. In this sub-study of the PermiT trial (Permissive Underfeeding or Standard Enteral Feeding in Critically Ill Adults Trial- ISRCTN68144998), we examined gene expression patterns in peripheral white blood cells (buffy coat) associated with moderate caloric restriction (permissive underfeeding) in critically ill patients compared to standard feeding. Blood samples collected on study day 1 and 14 were subjected to total RNA extraction and gene expression using microarray analysis. We enrolled 50 patients, 25 in each group. Among 1751 tested genes, 332 genes in 12 pathways were found to be significantly upregulated or downregulated between study day 1 and 14 (global p value for the pathway ≤ 0.05). Using the heatmap, the differential expression of genes from day 1 to 14 in the permissive underfeeding group was compared to the standard feeding group. We further compared gene expression signal intensity in permissive underfeeding compared standard feeding by constructing univariate and multivariate linear regression models on individual patient data. We found differential expression of several genes with permissive underfeeding, most notably those related to metabolism, autophagy and other cellular functions, indicating that moderate differences in caloric intake trigger different cellular pathways.

## Introduction

Evidence from recent randomized controlled trials and systematic reviews showed that short-term caloric restriction in the acute phase of critically ill patients was not associated with significant differences in mortality^1–6^, and might be associated with reduced bloodstream infections, acute kidney injury and mechanical ventilation duration^4,6^. Two randomized controlled trials found that delaying parenteral nutrition for 1 week was associated with improved outcomes in adult and pediatric critically ill patients^7,8^. The effect of short-term caloric restriction on gene expression in critically ill patients has not been studied. The objective of this study is to examine gene expression patterns in peripheral white blood cells associated with moderate caloric restriction (permissive underfeeding) in critically ill patients compared to standard feeding.

## Materials and Methods

### Study population

This is a pre-planned sub-study of the PermiT trial (Permissive Underfeeding versus Target Enteral Feeding in Adult Critically Ill Patients, Current Controlled Trials number, ISRCTN68144998)^1^. In the PermiT trial, critically ill patients were randomized to receive permissive underfeeding (40–60% of calculated caloric requirements) or standard feeding (70–100%) for up to 14 days with similar amount of protein intake provided to both groups. In this sub-study, patients enrolled in PermiT trial at King Abdulaziz Medical City, Riyadh, Saudi Arabia between September 2012 and August 2015 and expected to stay ≥14 days in the intensive care unit were consented for the study. The sub-study was approved by Institutional Board Review of the Ministry of the National Guard Health Affairs, Riyadh, Saudi Arabia and all clinical investigations were conducted according to the principles expressed in the Declaration of Helsinki.

### Nutrition

Caloric requirement was calculated using the Penn State equation for mechanically ventilated patients with body mass index (BMI) less than 30 kg/m^2^ and Ireton-Jones equation for mechanically ventilated patients with BMI of 30 kg/m^2^ or higher and for spontaneously breathing patients^9–11^. Protein target was uniform for both groups and was calculated as 1.2 to 1.5 g per kilogram of body weight per day. To provide similar protein to both groups, additional protein (Resource Beneprotein, Nestle Healthcare) was provided as needed. The intervention was continued up to 14 days, ICU discharge, initiation of oral feeding, death, or withholding of nutrition as part of palliation whichever came first.

### Clinical data collection

Baseline characteristics, intervention data and clinical outcome data were collected and compared between the two groups

### Blood sample analysis

Blood samples were collected on study day 1 and 14 and stored at −80 **°**C. Samples were subjected to total RNA extraction from peripheral white blood cells (buffy coat) using standard Promega kit (Cat # Z3100, Madison, WI) and evaluated with an Agilent 2100 Bioanalyzer (Agilent Technologies, Inc., Palo Alto, CA). RNA target was prepared following the Affymetrix manufacturer’s protocol (Affymetrix, Inc., Santa Clara, CA). Samples were then subjected to hybridization to GeneChips Affymetrix Human Exon 1.0 ST and scanned with a GeneChip 3000 High-Resolution Scanner (Affymetrix) that has over 5 million unique 25-mer oligonucleotides constituting 1.4 million probe sets and interrogating more than 150,000 transcripts. Data were extracted and analyzed using Expression Console software (Affymetrix) and GeneSpring v14.9 software (Agilent, Santa Clara, CA). Data from microarray experiments were preprocessed, background corrected and normalized per gene and chip and expressed as gene expression signal intensity.

### Statistical analysis

Continuous data were reported as medians and quartile 1, 3 (Q1, 3) and were compared using the Wilcoxon–Mann–Whitney test. Categorical variables were reported as numbers and frequencies. Analysis of Variance (ANOVA) was applied on the expressed probe sets and significant genes were filtered using global p-value of ≤0.05 for the pathway. The signal intensity of each probe was used to generate a heatmap using Genespring v14.9 software. Biological pathway search was performed on significantly expressed genes using WikiPathways, BioCyc genome databases and KEGG database. Using the heatmap, we evaluated the differential expression of genes from day 1 to 14 in the permissive underfeeding compared to the standard group as follows [(mean gene expression signal intensity in the permissive underfeeding group on day 14-day 1) − (mean gene expression signal intensity in standard feeding group on day 14-day 1)]. We further compared changes in gene expression from day 1 to 14 in the permissive underfeeding and standard feeding groups using individual patient values for gene expression signal intensity. We carried out univariate linear regression analysis to assess the differential gene expression signal intensity (day 14-day 1) in permissive underfeeding compared to standard feeding. To correct for age, the ratio of partial pressure of oxygen to the fraction of inspired oxygen (PaO2/FiO2) ratio and Sequential Organ Failure Assessment (SOFA) scores between the two groups, we carried out a multivariate linear regression analysis of the gene expression adjusting for these variables. We reported the results as β -coefficient and 95% confidence intervals (95% CI). Statistical significance was defined as p value of ≤0.05. Because of the limited existing data on the topic and the exploratory nature of the study, there was no formal sample size calculation performed. Clinical data was analyzed using SAS version 9.2 (SAS Institute, Cary, NC).

### Ethics approval

The study was approved by the Ministry of the National Guard Health Affairs Institutional Review Board (IRB), Riyadh, Saudi Arabia

### Consent to participants

An informed consent was obtained from these subjects for participating in the study procedures.

## Results

A total of 50 patients were enrolled in this study, 25 in each group (Supplement Fig. S1). Baseline characteristics were similar in both the groups including demographics, severity of illness and other physiological and biochemical parameters (Table 1). Nutritional intervention, co-interventions and outcomes are shown in Table 2 and Supplementary Table 1. Patients in the permissive underfeeding patients received fewer calories (median Q1, Q3- 990 (810.0, 1149.0) kcal/day) than the standard group (median Q1, Q3-1339.1 (1136.1, 1695.1) kcal/day), p value = 0.004. Total daily protein intake in the two groups was similar (median Q1, Q3-66.9 (47.0, 74.5) g/day) in permissive underfeeding vs (median Q1, Q3-61.1 (48.5, 75.1) g/day) in the standard group, p value 0.37.

**Table 1:** Baseline Characteristics of patients in the permissive underfeeding and standard feeding groups.

Variable	Permissive underfeeding n = 25	Standard feeding n = 25	P-value
Age (yrs), median (Q1, Q3)	28.0 (23.0, 58.1)	44.7 (27.5, 64.7)	0.21
Female sex, n (%)	4 (16.0)	4 (16.0)	1.00
Height (cm), median (Q1, Q3)	170 (164, 180)	170 (165, 173)	0.45
Weight (kg), median (Q1, Q3)	78 (54, 90)	82 (70, 88)	0.32
BMI (kg/m^2^), median (Q1, Q3)	27.2 (21.9, 33.2)	29.4 (24.8, 32.3)	0.46
Diabetes, n (%)	7 (28.0)	8 (32.0)	0.78
Inclusion blood glucose (mmol/L), median (Q1, Q3)	8.7 (7.6, 13.1)	9.0 (6.6, 11.3)	0.68
Admission category, n (%)			
Medical	10 (40.0)	9 (36.0)	0.88
Surgical	2 (8.0)	3 (12.0)	
Post-operative trauma	13 (52.0)	13 (52.0)	
APACHE II, median (Q1, Q3)	20.0 (14.0, 21.5)	16 (13, 26)	0.76
Mechanical ventilation, n (%)	25 (100)	25 (100)	1.00
Sepsis on admission, n (%)	3 (12.0)	2 (8.0)	0.64
Vasopressor, n (%)	18 (72.0)	15 (60.0)	0.37
Hemoglobin (g/L), median (Q1, Q3)	110 (97, 124)	111 (97, 128)	0.44
INR, median (Q1, Q3)	1.2 (1.0, 1.3)	1.1 (1.0, 1.3)	0.78
SOFA Score Day 1, median (Q1, Q3)	10.0 (8.5, 11.5)	11 (10, 12)	0.10
PaO2: FiO2 ratio, median (Q1, Q3)	211 (110, 286)	115 (88, 200)	0.11
Platelets (×10^9^/L), median (Q1, Q3)	172 (153, 264)	193 (176, 217)	0.53
Bilirubin, (μmol/L), median (Q1, Q3)	11.8 (8.7, 27.7)	19.0 (10.0, 29.1)	0.44
GCS, median (Q1, Q3)	3 (3, 4)	3 (3, 5)	0.61
Creatinine, (µmol/L), median (Q1, Q3)	77 (74, 114)	79 (69, 114)	0.76
C-reactive protein (mg/liter), median (Q1, Q3)	156.5 (111.5, 199.0)	132.0 (83.9, 187.0)	0.54
Serum lipid profile (mmol/liter), median (Q1, Q3)			
Cholesterol	2.5 (1.8, 2.9)	2.6 (2.0, 3.1)	0.50
Triglycerides	1.3 (1.0, 1.7)	1.4 (0.9, 2.0)	0.70
HDL	0.5 (0.4, 0.6)	0.5 (0.3, 0.6)	0.43
LDL	0.9 (0.7, 1.4)	1.1 (0.8, 1.5)	0.55
Albumin (g/L), median (Q1, Q3)	28.0 (24.5, 34.5)	28 (25, 33)	0.64
Pre-albumin (g/L), median (Q1, Q3)	0.12 (0.09, 0.13)	0.11 (0.08, 0.15)	0.88
Hemoglobin A1c, median (Q1, Q3)	0.06 (0.05, 0.06)	0.06 (0.06, 0.07)	0.33
24 hours urinary nitrogen excretion (mmol/L), median (Q1, Q3)	234 (74, 321)	274 (158, 381)	0.23
Transferrin (g/L), median (Q1, Q3)	1.2 (1.0, 1.5)	1.4 (1.2, 1.7)	0.03
Minute ventilation (L), median (Q1, Q3)	9.9 (8.4, 10.8)	10.2 (8.2, 11.3)	0.93
Maximum temperature (^0^C), median (Q1, Q3)	37.1 (36.7, 37.6)	37.3 (36.8, 37.9)	0.48

BMI: body mass index; APACHE II: Acute Physiology and Chronic Health Evaluation II; INR: international normalized ratio; SOFA: Sequential Organ Failure Assessment; GCS: Glasgow coma scale PaO2:FiO2 ratio: the ratio of partial pressure of oxygen to the fraction of inspired oxygen; HDL: High density lipoproteins; LDL: Low density lipoproteins.

The denominators for all percentages is the N for each column. Continuous variables are represented as median (quartile 1 and quartile 3).

**Table 2:** Daily caloric intake, protein intake, insulin, and glucose data in two groups.

Variable	Permissive underfeeding n = 25	Standard feeding n = 25	P-value
Calculated caloric requirement (kcal/day), median (Q1, Q3)	1746 (1585, 2075)	1909 (1770, 2162)	0.25
Study caloric target (kcal/day) median (Q1, Q3)	1071 (960, 1264)	1909 (1770, 2162)	<0.0001
Daily caloric intake			
No. of kilocalories, median (Q1, Q3)	990 (810.0, 1149.1)	1339.1 (1136.1, 1695.1)	0.004
Percent of requirement, median (Q1, Q3)	56.5 (51.1, 58.5)	67.6 (55.7, 89.6)	0.006
Caloric source (kcal/day) median (Q1, Q3)			
Enteral	936.9 (7.9.3, 1018.4)	1239.6 (1101.6, 1518.0)	0.01
Propofol	85.4 (11.0, 141.8)	78.6 (38.5, 232.7)	0.45
Intravenous dextrose	0.0 (0.0, 17.9)	0.0 (0.0, 29.10	0.51
Total parenteral nutrition	0 (0, 0)	0 (0, 0)	1.0
Calculated protein requirement (g/day) median (Q1, Q3)	84 (70, 95)	88 (82, 97)	0.16
Daily total protein intake			
No. of grams	66.9 (47.0, 74.5)	61.1 (48.5, 75.1)	0.37
Percent of requirement	82.1 (69.0, 88.8)	66.3 (50.3, 83.6)	0.07
Protein source (g/day), median (Q1, Q3)			
Main enteral formula	30.4 (25.0, 38.1)	51.8 (32.5, 64.5)	0.0007
Supplemental enteral protein	30.7 (22.3, 39.8)	0.0 (0.0, 6.1)	<0.0001
Parenteral protein	0 (0, 0)	0 (0, 0)	0.34
Duration of intervention (days) median (Q1, Q3)	13 (7, 14)	14 (8, 14)	0.33
Co-interventions			
Insulin			
Use, no. (%)	10 (40.0)	12 (48.0)	0.68
Dose (units/day), median (Q1, Q3)	0.0 (0.0, 23.4)	0.0 (0.0, 20.6)	0.47
Blood glucose (mmol/liter), median (Q1, Q3)	7.7 (6.6, 11.8)	8.4 (7.0, 11.0)	0.85
Enteral formulae on day 1^†^, n. (%)			
Disease-non-specific	15 (60.0)	13 (52.0)	0.59
Disease-specific	10 (40.0)	12 (48.0)	
Medications given during the ICU stay – n. (%)			
Beta blockers	10 (40.0)	11 (44.0)	0.77
Aspirin	4 (16.0)	5 (20.0)	0.82
Angiotensin-converting enzyme inhibitors	1 (4.0)	2 (8.0)	0.55
Angiotensin II receptor blockers	0 (0.0)	0 (0.0)	
Statins	5 (20.0)	4 (16.0)	0.71

^†^Disease-nonspecific formula: Osmolite, Jevity, Promote, Ensure plus, Resource, Ensure, Resource plus, Jevity (1.2).

^†^Disease-specific formula: Glucerna, Nutric hepatic, Nepro, Pulmocare, Novasource Renal, Peptamen (1.0), Peptamen (1.2), Suplena, Oxepa.

**Figure 1:**
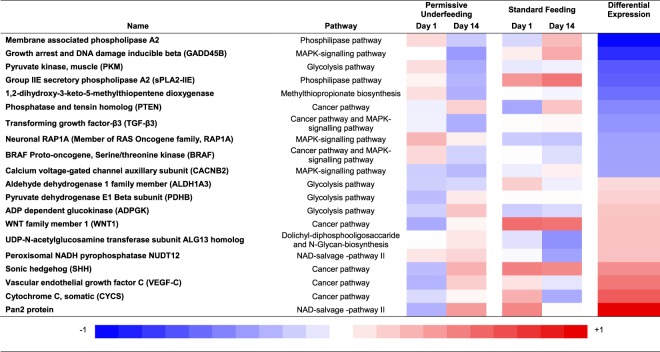
Gene expression in the permissive underfeeding and standard feeding groups. Blue-to-red colors indicates gene expression signal intensity from downregulated to upregulated based on p-value generated by built-in pathway module in GeneSpring software using the hypergeometric method. The differential expression (Diff. Expr.) is calculated as the difference in gene expression signal intensity in the permissive underfeeding group and the standard feeding group from day 1 to 14 [(mean gene expression signal intensity in the permissive underfeeding group on day 14-day 1) − (mean gene expression signal intensity in the standard feeding on day 14- day 1)]. Genes are sorted according to the ascending differential expression.

Among 1751 genes that were tested in the study cohort; 332 genes in 12 pathways were found to be significantly upregulated or downregulated between study day 1 and 14 (global p value for the pathway ≤0.05) (Table 3). The top 10 differentially downregulated or upregulated genes (Supplement Fig. S2) in the permissive underfeeding from day 1 to 14 compared to the standard feeding are presented in Fig. 1. The results of univariate and multivariate analyses are displayed in Table 4. Based on the multivariate analysis, the following genes were differentially downregulated in permissive underfeeding compared to standard feeding: membrane associated phospholipase A2, growth arrest and DNA damage inducible beta (GADD45B) and 1,2-dihydroxy-3-keto-5-methylthiopentene dioxygenase. On the other hand, the following genes were differentially upregulated with permissive underfeeding compared to standard feeding: somatic cytochrome C (CYCS), vascular endothelial growth factor C (VEGF-C), UDP-N-acetylglucosamine transferase subunit ALG13 homolog and ADP dependent glucokinase (ADPGK).

**Table 3:** Genes that were significantly upregulated or downregulated between study day 1 and 14 (global p value for the pathway ≤ 0.05).

Pathway	Global p-value	Number of up- or downregulated genes	Total number of genes in the pathway
Cancer pathways	0.00002	23	327
Glycolysis/Gluconeogenesis	0.00031	8	66
Phospholipase	0.00227	5	38
Salvage pathways of pyrimidine deoxyribonucleotides	0.00641	2	5
Dolichyl-diphosphooligosaccharide biosynthesis	0.01704	2	8
Uracil degradation II (reductive)	0.01704	2	8
Thymine degradation	0.01704	2	8
Methylthiopropionate biosynthesis	0.02600	1	1
Pentose phosphate pathway	0.02647	2	10
MAPK signaling pathway	0.03237	12	257
N-Glycan biosynthesis	0.03827	4	49
NAD salvage pathway II	0.04358	2	13

**Table 4:** Comparison of changes in gene expression signal intensity from day 1 to 14 in the permissive underfeeding and standard feeding groups.

Gene Name	Change in gene expression (Day 14-Day 1)		Univariate analysis			Multivariate analysis		
	Permissive feeding	Standard feeding	β -coefficient	95% CI	P value	β -coefficient	95% CI	P value
**Differentially downregulated gene expression with permissive underfeeding**								
Membrane-associated phospholipase A2	−0.086 ± 0.192	0.102 ± 0.206	−0.188	−0.303, −0.074	0.002	−0.194	−0.319, −0.069	0.003
Growth arrest and DNA damage inducible beta (GADD45B)	−0.098 ± 0.186	0.066 ± 0.287	−0.163	−0.303, −0.023	0.023	−0.156	−0.303, −0.009	0.038
Pyruvate kinase, muscle (PKM)	−0.087 ± 0.151	0.007 ± 0.164	−0.094	−0.185, −0.003	0.043	−0.085	−0.184, 0.014	0.091
Group IIE secretory phospholipase A2 (sPLA2-IIE)	−0.090 ± 0.218	0.033 ± 0.411	−0.124	−0.314, 0.067	0.198	−0.139	−0.347, 0.070	0.186
1,2-dihydroxy-3-keto-5-methylthiopentene dioxygenase	−0.106 ± 0.141	−0.008 ± 0.192	−0.097	−0.194, −0.0004	0.049	−0.115	−0.221, −0.010	0.033
Phosphatase and tensin homolog (PTEN)	0.042 ± 0.279	0.135 ± 0.271	−0.093	−0.251, 0.065	0.243	−0.111	−0.280, 0.057	0.190
Transforming growth factor-β3 (TGF-β3)	−0.052 ± 0.126	0.004 ± 0.110	−0.056	−0.124, 0.011	0.102	−0.057	−0.131, 0.016	0.120
Neuronal RAP1A (Member of RAS Oncogene family, RAP1A)	−0.063 ± 0.266	−0.006 ± 0.169	−0.057	−0.185, 0.070	0.372	−0.031	−0.166, 0.103	0.641
BRAF Proto-oncogene, Serine/threonine kinase (BRAF)	−0.083 ± 0.146	−0.028 ± 0.146	−0.055	−0.139, 0.029	0.194	−0.068	−0.160, 0.024	0.144
Calcium voltage-gated channel auxillary subunit (CACNB2)	−0.008 ± 0.131	0.031 ± 0.151	−0.038	−0.120, 0.043	0.348	−0.061	−0.147, 0.025	0.160
**Differentially upregulated gene expression with permissive underfeeding**								
Aldehyde dehydrogenase 1 family member (ALDH1A3)	0.012 ± 0.135	−0.062 ± 0.139	0.074	−0.004, 0.153	0.064	0.080	−0.008, 0.167	0.072
Pyruvate dehydrogenase E1 Beta subunit (PDHB)	0.064 ± 0.164	0.002 ± 0.173	0.063	−0.034, 0.160	0.199	0.042	−0.064, 0.147	0.431
ADP dependent glucokinase (ADPGK)	0.107 ± 0.164	0.011 ± 0.149	0.096	0.006, 0.186	0.037	0.105	0.007, 0.204	0.036
WNT family member 1 (WNT1)	0.111 ± 0.260	−0.006 ± 0.227	0.117	−0.023, 0.258	0.099	0.125	−0.024, 0.273	0.098
UDP-N-acetylglucosamine transferase subunit ALG13 homolog	0.013 ± 0.166	−0.068 ± 0.137	0.081	−0.006, 0.169	0.067	0.100	0.008, 0.192	0.033
Peroxisomal NADH pyrophosphatase NUDT12	0.007 ± 0.206	−0.077 ± 0.212	0.084	−0.036, 0.205	0.164	0.050	−0.078, 0.178	0.433
Sonic hedgehog (SHH)	0.131 ± 0.244	−0.007 ± 0.301	0.139	−0.019, 0.297	0.084	0.167	−0.006, 0.340	0.058
Vascular endothelial growth factor C (VEGF-C)	0.111 ± 0.228	−0.052 ± 0.155	0.163	0.051, 0.274	0.005	0.162	0.044, 0.280	0.008
Cytochrome C, somatic (CYCS)	0.019 ± 0.304	−0.152 ± 0.246	0.171	0.013, 0.330	0.035	0.176	0.003, 0.349	0.046
Pan2 protein	0.132 ± 0.433	−0.118 ± 0.340	0.249	0.026, 0.472	0.029	0.182	−0.041, 0.406	0.108

Univariate analysis and multivariate linear regression models were carried out to assess the gene expression signal intensity in permissive underfeeding compared to standard feeding. In the multivariate model, we adjusted for age, the ratio of partial pressure of oxygen to the fraction of inspired oxygen (PaO2/FiO2) ratio and Sequential Organ Failure Assessment (SOFA) scores.

## Discussion

We found differential downregulation and upregulation of several genes related to metabolism with permissive underfeeding compared to standard feeding.

The muscle pyruvate kinase (PKM) gene, which is known to be stimulated by carbohydrates^12^, was downregulated with permissive underfeeding and upregulated with standard feeding. The differential expression of PMK was statistically significant on univariate analysis of individual patient data (p 0.042) and borderline on multivariate analysis (p 0.091). ADP dependent glucokinase (ADPGK) gene was also differentially upregulated with permissive underfeeding compared to standard feeding (differential expression by multivariate analysis of individual patient data p 0.049). ADP-dependent glucokinase (ADPGK) catalyzes glucose-6-phosphate production, utilizing ADP as a phosphoryl donor. ADPGK has been shown to be substrate-inhibited by high glucose concentration, which may explain its differential upregulation with permissive underfeeding compared to standard feeding. The somatic cytochrome C (CYSC) gene was upregulated with permissive underfeeding and downregulated with standard feeding, the differential expression was significant (differential expression by multivariate analysis of individual patient data p 0.046). CYSC functions as a central component of the respiratory chain in mitochondria and is involved in initiation of apoptosis. Interestingly, low protein supply during gestation in porcine model was associated with large increase in CYSC gene expression in the liver on day 1 post natum; while high protein supply was associated with only a slight increase in CYSC gene expression^13^.

In addition, we found that permissive underfeeding compared to standard feeding was associated with downregulation and upregulation of several genes related to autophagy. The Growth arrest and DNA damage inducible beta (GADD45B) gene was downregulated with permissive underfeeding and upregulated with standard feeding (differential expression by multivariate analysis of individual patient data p 0.038). The GADD45B protein is believed to play a role in preventing autophagy and apoptosis^14,15^, suggesting that permissive underfeeding stimulates and standard feeding inhibits autophagy. In addition, permissive underfeeding was differentially associated with upregulation of the vascular endothelial growth factor C (VEGF-C) gene (differential expression by multivariate analysis of individual patient data p 0.008). VEGF-C functions as a specific growth factor for lymphatic vessels, promotes the growth of blood vessels and regulates their permeability. The VEGF-C is involved in the activation of autophagy in cancer cells promoting their survival^16,17^, probably through inhibition of the mammalian target of rapamycin (mTOR) complex 1 activity^17^. Recent evidence suggests that autophagy is an important repair process for recovery from organ dysfunction in critically ill patients^18^. Our findings are in line of the results of a sub-study of the Early Parenteral Nutrition Completing Enteral Nutrition in Adult Critically Ill Patients (EPaNIC) trial^7,19^. On muscle biopsies, the LC3 (microtubule-associated protein light chain 3) II to LC3I ratio, which related to autophagosome formation, was higher in patients given late PN than early PN and was independently associated with less weakness. The study suggested that delaying PN allowed more efficient activation of autophagic quality control of myofibres and reduced weakness^19^.

Additionally, there were genes associated with inflammation, cell proliferation and apoptosis that were differentially expressed with permissive underfeeding compared to standard feeding. Membrane-associated phospholipase A2, which is involved in eicosanoid biosynthesis, was downregulated in permissive underfeeding and upregulated in standard feeding (differential expression by multivariate analysis of individual patient data, p 0.003).

In addition, several genes were found to be differentially expressed on heatmap which reflects mean gene expression signal intensity for the group, however, this was not maintained in univariate or multivariate analyses on individual patient values. This may be related to the study sample size. The Group IIE secretory phospholipase A2 gene (sPLA2-IIE gene) was downregulated with permissive underfeeding and minimally changed with standard feeding on heatmap, although the differential expression did not reach statistical significance on individual patient data analysis. The sPLA2-IIE gene has an important role in inflammation^20^; its expression is highly induced in mice injected with lipopolysaccharide and is associated with increase in leukotriene production^21^. sPLA2-IIE is also has a metabolic role^22^; its expression is upregulated in adipocytes of obese mice and is associated with adiposity and fatty liver^23^. Similarly, transforming growth factor-β3 gene (TGF-β3) was downregulated with permissive underfeeding and upragulated with standard feeding on heatmap, although the differential expression did not reach statistical significance on individual patient data analysis. TGF-β3 is a cytokine that is involved in embryogenesis, cell differentiation and wound healing^24^. A study found that TGF-β genes, including TGF-β3, were associated with the risk of metabolic syndrome among Taiwanese individuals^25^. The neuronal RAP1A gene was downregulated in both permissive and standard feeding group on heatmap, although downregulation in the permissive underfeeding was more profound, but the differential expression was not statistically significant on individual patient data analysis. The RAP1A gene is expressed in multiple hypothalamic nuclei that control whole-body metabolism and is activated in high-fat diet induced obesity^26^. Genetic ablation or pharmacologic inhibition of neuronal RAP1A gene in mice reduces insulin resistance, improves leptin sensitivity in the hypothalamus and protects from dietary obesity^26^. The WNT1 gene was upregulated with permissive underfeeding but was almost unchanged with standard feeding on heatmap but the differential expression was not statistically significant on individual patient data analysis. The WNT1 gene is involved in lipid metabolism and obesity development^27^. Feeding obesity-prone rats with high-fat diet was associated with lower expression of WNT1 gene, lower expression of insulin receptor substrate and higher body weight and blood triglyceride levels than obesity resistant rats^27^. In another study, offspring of pregnant rats that were fed with high-fat diet during gestation and lactation had increased serum glucose and liver triglyceride levels associated with downregulation of WNT1 gene expression^28^.

Permissive underfeeding was associated with slight upregulation and standard feeding with substantial upregulation of the phosphatase and tensin homolog (PTEN) gene on heatmap, although the differential expression did not reach statistical significance on individual patient data analysis. PTEN gene functions as a tumor suppressor gene and has a role in inducing apoptosis, but its function during critical illness is unclear. However, our findings are in line with a study that found that high glucose levels was associated with a significant concentration-dependent upregulation of PTEN gene in human vascular endothelial cells with decreased cell viability, induced apoptosis, and elevated levels of intracellular reactive oxygen species^29^. BRAF was downregulated in both permissive and standard feeding group on heatmap, although downregulation in the permissive underfeeding was more profound and the differential expression did not reach statistical significance on individual patient data analysis. BRAF is a proto-oncogene that is frequently mutated in colorectal cancer. A study showed that dietary fat promotes mutated BRAF (BRAF V600E) tumor growth and that hypolipidemic agents inhibit BRAF V600E tumor growth^30^. The Sonic hedgehog gene was upregulated differentially in the permissive underfeeding group on heatmap, but not on individual patient data analysis; this gene controls cell division of adult stem cells, but its role during critical illness is unclear.

To our knowledge this is the first study to assess the influence of moderate short-term caloric restriction on gene expression in critically ill patients. The strengths of the study include being part of a randomized controlled trial, which minimizes the imbalances in the characteristics of patients between the two groups. Nevertheless, because of small, albeit not statistically significant differences in age, SOFA scores and PaO2/FiO2 ratio, we carried multivariate analyses adjusting for these variables to account for these imbalances. We had a baseline and day 14 expression studies, which allows evaluating the changes over time and the use of each patient as his own control. Protein intake was similar by design of the study, which allowed isolated caloric difference to be the main exposure. The study examined gene expression patterns in peripheral white blood cells, and some of the changes may or may not reflect gene expression in other tissues. However, our study suggests the need for further tissue-specific gene expression studies.

## Conclusion

In conclusion, the present study shows that different caloric intake via enteral nutrition lead to differential expression of a wide variety in genes, most notably those related to metabolism, autophagy and other cellular functions, indicating that differences in caloric intake trigger different cellular pathways.

## Electronic supplementary material

Supplementary Information

## Data Availability

The datasets used and/or analyzed during the current study are available from the corresponding author on reasonable request.
