# Supplementary material for: Differential Gene Expression in Peripheral White Blood Cells with Permissive Underfeeding and Standard Feeding in Critically Ill Patients: A Descriptive Sub-study of the PermiT Randomized Controlled Trial
Source: Sci Rep. 2018 Dec 20;8:17984. doi: 10.1038/s41598-018-36007-w (PMC6301949; doi:10.1038/s41598-018-36007-w)
Supplement: Supplementary file 1 — Supplementary Information [file 41598_2018_36007_MOESM1_ESM.doc]

**Online Supplement**

**Differential Gene Expression in Peripheral White Blood Cells with Permissive Underfeeding and Standard Feeding in Critically Ill Patients: A Descriptive Sub-study of the PermiT Randomized Controlled Trial**

**Yaseen. M Arabi**, Mohammed Al-Balwi, Ali H. Hajeer, Dunia Jawdat, Musharaf Sadat, Hasan M. Al-Dorzi, Hani Tamim, Lara Afesh, Walid Mashaqbeh, Haitham Alkadi, Deemah Alwadaani, UdayaRaja.G.K, Ibrahim B Al Abdulkareem, Abdulaziz Al-Dawood.

.

**Supplementary Table 1:** Outcomes in the permissive underfeeding and standard feeding groups.

| **Outcomes** | **Permissive underfeeding**  **n = 25** | **Standard feeding**  **n =25** | **P value** | **OR (95%CI)** |
| --- | --- | --- | --- | --- |
|  |  |  |  |  |
| Death by 28 days, n. (%) | 1 (4.0) | 3 (12.0) | 0.30 | 0.31 (0.03, 3.16) |
| Death by 90 days, n. (%) | 2 (8.0) | 3 (20.0) | 0.27 | 0.40 (0.08, 2.13) |
| Death by 180 days, n. (%) | 2 (8.3) | 5 (20.0) | 0.24 | 0.36 (0.06, 2.09) |
| Death in the ICU, n. (%) | 0 (0.0) | 2 (6.7) | 0.15 | - |
| New renal replacement therapy, n. (%) | 2 (8.3) | 5 (20.0) | 0.24 | 0.36 (0.06, 2.09) |
| ICU length of stay (days), median (Q1, Q3) | 17 (10, 28) | 21 (15, 28) | 0.44 |  |
| Hospital length of stay (days), median (Q1, Q3) | 72 (32, 112) | 46 (34, 109) | 0.62 |  |
| Duration of mechanical ventilation (days), median (Q1, Q3) | 12 (8, 16) | 17 (11, 22) | 0.11 |  |
|  |  |  |  |  |

ICU: intensive care unit

**Supplement Figure 1.** Flow diagram for patients enrolled in the study.

**Supplement Figure S2:** Gene expression in the permissive underfeeding and standard feeding groups. Blue-to-red colors indicate expression levels from downregulated to upregulated based on p-value generated by built-in pathway module in GeneSpring Software using the hypergeometric method. The differential expression (Diff. Expr.) is calculated as the difference in gene expression level in the permissive underfeeding group and the standard feeding group from day 1 to 14 [(Permissive underfeeding day 14-1) – (Standard feeding day 14-1). Genes are sored according to the differential expression.

| **Name** | **Pathway** | **Permissive underfeeding** | | **Standard feeding** | | |  | **Diff. expr** |
| --- | --- | --- | --- | --- | --- | --- | --- | --- |
|  |  | **Day**  **1** | **Day**  **14** |  | **Day 1** | **Day 14** |  |  |
| **Membrane associated phospholipase A2** | Phospholipase pathway |  |  |  |  |  |  |  |
| **Growth arrest and DNA damage inducible beta (GADD45B)** | MAPK-signaling pathway |  |  |  |  |  |  |  |
| **Pyruvate kinase, muscle (PKM)** | Glycolysis pathway |  |  |  |  |  |  |  |
| **Group IIE secretory phospholipase A2** | Phospholipase pathway |  |  |  |  |  |  |  |
| **1,2-dihydroxy-3-keto-5-methylthiopentene dioxygenase** | Methylthiopropionate biosynthesis |  |  |  |  |  |  |  |
| **Phosphatase and tensin homolog ( PTEN)** | Cancer pathway |  |  |  |  |  |  |  |
| **Transforming growth factor-beta3 (TGFB3)** | Cancer pathway |  |  |  |  |  |  |  |
| **Transforming growth factor-beta 3 (TGFB3)** | MAPK-signaling pathway |  |  |  |  |  |  |  |
| **Member of RAS Oncogene family (RAP1A)** | MAPK-signaling pathway |  |  |  |  |  |  |  |
| **BRAF Proto-oncogene, Serine/threonine kinase (BRAF)** | Cancer pathway |  |  |  |  |  |  |  |
| **BRAF Proto-oncogene, Serine/threonine kinase (BRAF)** | MAPK-signaling pathway |  |  |  |  |  |  |  |
| **Calcium voltage-gated channel auxiliary subunit (CACNB2)** | MAPK-signaling pathway |  |  |  |  |  |  |  |
| **Fibronectin 1 (FN1)** | Cancer pathway |  |  |  |  |  |  |  |
| **Calcium voltage-gated channel auxiliary subunit (CACNA2D4)** | MAPK-signaling pathway |  |  |  |  |  |  |  |
| **AKT Serine/threonine kinase 3 (AKT3)** | Cancer pathway |  |  |  |  |  |  |  |
| **AKT Serine/threonine kinase 3 (AKT3)** | MAPK-signaling pathway |  |  |  |  |  |  |  |
| **Fibroblast growth factor 2 (FGF2)** | Cancer pathway |  |  |  |  |  |  |  |
| **Fibroblast growth factor 2 (FGF2)** | MAPK-signaling pathway |  |  |  |  |  |  |  |
| **Activation-induced cytidine deaminase** | Savage-pathways of pyramidine |  |  |  |  |  |  |  |
| **Interleukin 6 (IL6)** | Cancer pathway |  |  |  |  |  |  |  |
| **Fibroblast growth factor 9 (FGF9)** | Cancer pathway |  |  |  |  |  |  |  |
| **Fibroblast growth factor 9 (FGF9)** | MAPK-signaling pathway |  |  |  |  |  |  |  |
| **CBL Proto-oncogene (BCBLB)** | Cancer pathway |  |  |  |  |  |  |  |
| **Dihydrolipoamide dehdrogenase (DLD)** | Glycolysis pathway |  |  |  |  |  |  |  |
| **S-Phase Kinase Associated Protein 2 (SKP2)** | Cancer pathway |  |  |  |  |  |  |  |
| **Fms related tyrosine kinase 3 ligand (FLT3LG)** | Cancer pathway |  |  |  |  |  |  |  |
| **Integrin subunit alpha 2 (ITGA2)** | Cancer pathway |  |  |  |  |  |  |  |
| **Phospholipase C Delta1** | Phospholipase pathway |  |  |  |  |  |  |  |
| **Group IID secretory phospholipase A2** | Phospholipase pathway |  |  |  |  |  |  |  |
| **Adaptor protein, phosphotyrosineInteracting With PH**  **Domain And Leucine Zipper (APPL1)** | Cancer pathway |  |  |  |  |  |  |  |
| **Protein phosphatase 1 (AXIN1)** | Cancer pathway |  |  |  |  |  |  |  |
| **laminin subunit beta 4 (LAMB4)** | Cancer pathway |  |  |  |  |  |  |  |
| **Breast cancer 2) BRCA2** | Cancer pathway |  |  |  |  |  |  |  |
| **Mannosidase Alpha Class 2A Member 1 (MAN2A1)** | N-Glycan-biosynthesis |  |  |  |  |  |  |  |
| **Alcohol dehydrogenase 6 class V (ADH6)** | Glycolysis pathway |  |  |  |  |  |  |  |
| **Glucose-6-phosphate 1-dehydrogenase** | Pentose-phosphate pathway |  |  |  |  |  |  |  |
| **mutS homolog 2 (MSH2)** | Cancer pathway |  |  |  |  |  |  |  |
| **Ribophorin II (RPN2)** | N-Glycan-biosynthesis |  |  |  |  |  |  |  |
| **Dihydropyrimidinase** | Thymidine-degradation-entities |  |  |  |  |  |  |  |
| **Dihydropyrimidinase** | Uracil degradation |  |  |  |  |  |  |  |
| **Acyl-CoA synthetase short chain family member (ACSS1)** | Glycolysis pathway |  |  |  |  |  |  |  |
| **Dolichyl pyrophosphate Man9GlcNAc2 alpha-1,3-**  **Glucosyltransferase** | Dolichyl-diphosphooligosaccaride |  |  |  |  |  |  |  |
| **Alpha-1, 3-Glucosyltransferase (ALG6)** | N-Glycan-biosynthesis |  |  |  |  |  |  |  |
| **TNF receptor associated factor 6 (TRAF6)** | Cancer pathway |  |  |  |  |  |  |  |
| **Transketolase-like protein 2** | Pentose-phosphate pathway |  |  |  |  |  |  |  |
| **Thymidine kinase, cytosolic** | Savage-pathways of pyramidine |  |  |  |  |  |  |  |
| **Dihydropyrimidinase-related protein 3** | Thymidine-degradation-entities |  |  |  |  |  |  |  |
|  | **Pathway** | **Permissive underfeeding** | |  | **Standard feeding** | |  | **Diff.**  **expr** |
| **Name** |  | **Day**  **1** | **Day**  **14** |  | **Day 1** | **Day 14** |  |  |
|  |  |  |  |  |  |  |  |  |
| **Dihydropyrimidinase-related protein 3** | Uracil degradation |  |  |  |  |  |  |  |
| **Phosphoglycerate kinase 2 (PGK2)** | Glycolysis pathway |  |  |  |  |  |  |  |
| **Protein phosphatase 3 catalytic subunit gamma (PPP3CC)** | MAPK-signaling pathway |  |  |  |  |  |  |  |
| **Mitogen-activated protein kinase 2 (MAP2K2)** | Cancer pathway |  |  |  |  |  |  |  |
| **Mitogen-activated protein kinase 2 (MAP2K2)** | MAPK-signaling pathway |  |  |  |  |  |  |  |
| **Phospholipase D4** | Phospholipase pathway |  |  |  |  |  |  |  |
| **Ribosomal protein S6 kinase A6 (RPS6KA6)** | MAPK-signaling pathway |  |  |  |  |  |  |  |
| **Aldehyde dehydrogenase 1 family member (ALDH1A3)** | Glycolysis pathway |  |  |  |  |  |  |  |
| **Pyruvate dehydrogenase E1 Beta subunit (PDHB)** | Glycolysis pathway |  |  |  |  |  |  |  |
| **ADP dependent glucokinase (ADPGK)** | Glycolysis pathway |  |  |  |  |  |  |  |
| **WNT family member 1 (WNT1)** | Cancer pathway |  |  |  |  |  |  |  |
| **UDP-N-acetylglucosamine transferase subunit ALG13 homolog** | Dolichyl-diphosphooligosaccaride |  |  |  |  |  |  |  |
| **UDP-N-Acetylglucosaminyltransferase Subunit (ALG13)** | N-Glycan-biosynthesis |  |  |  |  |  |  |  |
| **Peroxisomal NADH pyrophosphatase NUDT12** | NAD-salvage -pathway II |  |  |  |  |  |  |  |
| **Sonic hedgehog (SHH)** | Cancer pathway |  |  |  |  |  |  |  |
| **Vascular endothelial growth factor C (VEGFC)** | Cancer pathway |  |  |  |  |  |  |  |
| **Cytochrome c, somatic (CYCS)** | Cancer pathway |  |  |  |  |  |  |  |
| **Pan2 protein** | NAD-salvage -pathway II |  |  |  |  |  |  |  |
| -1 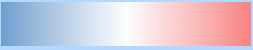 +1 | | | | | | | | |
